# Ventilation Onset Prior to Umbilical Cord Clamping (Physiological-Based Cord Clamping) Improves Systemic and Cerebral Oxygenation in Preterm Lambs

**DOI:** 10.1371/journal.pone.0117504

**Published:** 2015-02-17

**Authors:** Graeme R. Polglase, Jennifer A. Dawson, Martin Kluckow, Andrew W. Gill, Peter G. Davis, Arjan B. te Pas, Kelly J. Crossley, Annie McDougall, Euan M. Wallace, Stuart B. Hooper

**Affiliations:** 1 The Ritchie Centre, Monash Institute of Medical Research—Prince Henrys Institute, Clayton, Victoria; 2 Department of Obstetrics and Gynaecology, Monash University, Clayton, Victoria; 3 Newborn Research Centre, Royal Women's Hospital, Melbourne, Victoria; 4 Department of Neonatology, Royal North Shore Hospital and University of Sydney, Sydney, New South Wales; 5 Centre for Neonatal Research and Education, The University of Western Australia, Western Australia, Australia; 6 Division of Neonatology, Department of Pediatrics, Leiden University Medical Center, Leiden, The Netherlands; Icahn School of Medicine at Mount Sinai, ARGENTINA

## Abstract

**Background:**

As measurement of arterial oxygen saturation (SpO_2_) is common in the delivery room, target SpO_2_ ranges allow clinicians to titrate oxygen therapy for preterm infants in order to achieve saturation levels similar to those seen in normal term infants in the first minutes of life. However, the influence of the onset of ventilation and the timing of cord clamping on systemic and cerebral oxygenation is not known.

**Aim:**

We investigated whether the initiation of ventilation, prior to, or after umbilical cord clamping, altered systemic and cerebral oxygenation in preterm lambs.

**Methods:**

Systemic and cerebral blood-flows, pressures and peripheral SpO_2_ and regional cerebral tissue oxygenation (SctO_2_) were measured continuously in apnoeic preterm lambs (126±1 day gestation). Positive pressure ventilation was initiated either 1) prior to umbilical cord clamping, or 2) after umbilical cord clamping. Lambs were monitored intensively prior to intervention, and for 10 minutes following umbilical cord clamping.

**Results:**

Clamping the umbilical cord prior to ventilation resulted in a rapid decrease in SpO_2_ and SctO_2_, and an increase in arterial pressure, cerebral blood flow and cerebral oxygen extraction. Ventilation restored oxygenation and haemodynamics by 5–6 minutes. No such disturbances in peripheral or cerebral oxygenation and haemodynamics were observed when ventilation was initiated prior to cord clamping.

**Conclusion:**

The establishment of ventilation prior to umbilical cord clamping facilitated a smooth transition to systemic and cerebral oxygenation following birth. SpO_2_ nomograms may need to be re-evaluated to reflect physiological management of preterm infants in the delivery room.

## Introduction

Aeration of the lungs at birth is the primary trigger for the transition from fetal to newborn patterns of gas exchange and cardiopulmonary circulation[1–4]. While the majority of infants manage this transition without any difficulty, the most vulnerable infants, particularly those born preterm, often require assistance in the form of oxygen therapy or respiratory support to facilitate this transition. Indeed, 18.7% of all babies born in Australia in 2009 required respiratory support in the form of oxygen therapy or intermittent positive pressure ventilation to assist with the transition at birth[5]. Clinicians have used pulse oximetry measurements of heart rate and peripheral oxygen saturation to guide intervention.

Dawson et al., 2010 reported the normal range of SpO_2_ in preterm infants during the first 10 minutes of life[6]. These infants all had the umbilical cord clamped early, which was the standard practice at the time. These nomograms showed that the median SpO_2_ at 1 minute is 62% (47–72% IQR) for preterm infants and 68% (55–75%) for term infants not requiring respiratory support. Therefore, half of these infants have an SpO_2_ lower than normal fetal levels at 1 min, with infants delivered by caesarean section having significantly lower SpO_2_ levels than vaginally delivered infants[7–9]. Similar nomograms have been established for cerebral regional oxygenation (SctO_2_) in newborn infants; the median (10th-90th percentiles) SctO_2_ was 41% (23–64) at 2 minutes, and increasing to 79% (65–90) at 10 minutes[10]. An important determinate of the initial SpO_2_ and SctO_2_ may be whether adequate respiration has been established prior to umbilical cord clamping.

The timing of umbilical cord clamping during the fetal newborn transition has received renewed attention recently. Studies have shown that delayed cord clamping (DCC) has many immediate, short-term and long-term benefits in preterm infants, including improved cardiopulmonary adaptation[11,12], reduced need for blood transfusions, and decreased incidence of intraventricular haemorrhage and necrotising enterocolitis[7,13]. The major mechanism responsible for the benefits of DCC was thought to be increased placental transfusion resulting in greater blood volume[7,14]. However, we have recently shown that the initiation of ventilation prior to cord clamping may be more important than the delay in cord clamping itself[15]. This study demonstrated that the initiation of ventilation prior to cord clamping improved cardiac output by increasing pulmonary blood flow before the cord is clamped. This enables pulmonary venous return to immediately replace umbilical venous return as the primary source of left ventricular output, without any reduction in supply, thereby stabilising the cerebral haemodynamic transition. This effect is consistent with recent clinical trials showing that delayed cord clamping improves cardiac output after birth[16,17]. However, the interaction between the timing of ventilation onset relative to umbilical cord clamping on systemic and cerebral oxygenation is not known.

The establishment of adequate ventilation of preterm infants is not only critical to trigger the transition from fetal to newborn physiology, but it also ensures provision of adequate oxygen delivery to important organs, especially the brain. The aim of this study was to compare systemic and cerebral oxygenation in preterm lambs when ventilation was initiated prior to, or after umbilical cord clamping. We hypothesized that aerating the lung and therefore providing an alternative source of oxygenation prior to removal of the placental circulation with umbilical cord clamping, would improve systemic and cerebral oxygenation during the first 10 min after birth.

## Methods

### Ethics Statement

The experimental protocol was performed in accordance with guidelines established by the National Health and Medical Research Council of Australia and was approved by the relevant animal ethics committee at Monash University.

### Experimental Design

At 126 ± 2 days gestation (term ∼ 148 days), Border-Leicester ewes were anaesthetised with an intravenous bolus of 5% sodium thiopentone (Pentothal; 1g in 20 ml) and, following intubation, maintained with inhalation of 1.5–3% halothane in air. The fetal head and neck were exposed via caesarean section and an ultrasonic flow probe (3 mm: Transonic Systems, Ithaca, NY, USA) was placed around a carotid artery. Heparinised saline-filled polyvinyl catheters were inserted into the other carotid artery and into a jugular vein. The fetal trachea was intubated with a 4.0 mm cuffed endotracheal tube and lung liquid was drained passively for ∼ 10 seconds or until liquid ceased exiting the airways. A transcutaneous arterial oxygen saturation (SpO_2_) probe (Masimo, Radical 4, CA, USA) was placed around the right forelimb and the output recorded continuously. A Near Infrared Spectroscopy optode (Casmed Foresight, CAS Medical Systems Inc, Branford, CT, USA) was placed over the left frontal cortex and used to continuously measure cerebral tissue oxygen saturation (SctO_2_). After completion of instrumentation, the ewe was rotated onto its side and the fetus was completely exteriorised from the uterus, still attached to the umbilical cord, dried, and placed on a delivery table immediately next to the ewe. Physiological parameters were allowed to stabilise prior to the birth procedures being initiated (see below)—the delay between exteriorisation and intervention was a mean (SD) of 181 ± 31 seconds for all lambs.

Each fetus was randomised to either umbilical cord clamping prior to initiation of ventilation (Clamp 1^st^; n = 10) or the initiation of ventilation prior to umbilical cord clamping (Vent 1^st^; n = 7) groups. In Clamp 1^st^ lambs, the umbilical cord was immediately clamped and cut, the lamb transferred to an infant warmer (CosyCot, Fisher and Paykel, Auckland, New Zealand) and ventilation commenced as soon as possible. In Vent 1^st^ lambs, ventilation commenced while the umbilical cord remained patent. Umbilical cord clamping was delayed until after PBF had increased, indicating cardiopulmonary transition, whereupon the cord was clamped and cut. In both groups ventilation was initiated using positive pressure ventilation in volume guarantee mode with a tidal volume of 7 mL/kg and a positive end-expiratory pressure of 5 cmH_2_O using warmed and humidified inspired gases, with an initial fraction of inspired oxygen (FiO_2_) of 21% (Babylog 8000+, Dräger, Lübeck, Germany). Peak inspiratory pressure was limited to 40 cmH_2_O to avoid pneumothoraces. FiO_2_ was adjusted to target SpO_2_ at 70–90% by 3 min, 80–90% by 5 min and 90–96% by 10 min.

Regular blood gas analysis (ABL30, Radiometer, Copenhagen, Denmark) and real-time SpO_2_ measurements were used to monitor the lambs. All lambs received sedation (Alfaxane i.v. 5–15 mg/kg/h; Jurox, East Tamaki, Auckland, New Zealand) in 5% dextrose via the jugular vein catheter to minimize spontaneous breathing during the experiment. The ewes were humanely euthanized using sodium pentobarbitone (100 mg/kg i.v) after caesarean section and the lambs were euthanized after completion of the ventilation study, at 30 min—2 h later, depending on the study.

### Measurements

Instantaneous blood flow in the carotid artery, used as a proxy for cerebral blood flow [18] (CBF), SaO_2_ and SctO_2_ were recorded digitally using a data acquisition system (Powerlab; ADInstruments, Castle Hill, Australia). Arterial pressures were measured using pressure transducers (PD10; DTX Plus Transducer; Becton Dickinson, Singapore) and also recorded digitally.

### Calculations

SpO_2_ and SctO_2_ were used to calculate cerebral oxygen consumption and extraction as described previously[19]; Cerebral oxygen consumption (VO_2_) was calculated as: Mean CBF x (SaO_2_-SctO_2_) where CBF = cerebral blood flow, measured from the carotid artery.

Cerebral oxygen extraction was calculated as: SaO_2_-SctO2/SaO_2_.

Arterial blood gas samples prior to intervention (i.e. fetal sample) and at 5 and 10 minutes after cord clamping were used to calculate oxygenation index, arterial oxygen content and cerebral oxygen delivery: Oxygenation Index = (F_i_O_2_ x mean airway pressure)/PaO_2_, where FiO_2_ is the inspired oxygen concentration and PaO_2_ is the arterial oxygen tension.

Arterial oxygen content (CaO_2_) = [1.39•Hb•SaO_2_/100] + [0.003•PaO_2_])[20], where Hb is the haemoglobin concentration (g/dL).

Cerebral oxygen delivery (DO_2_) = (CBF•CaO_2_)[20].

### Statistics

Fetal data collected prior to intervention were compared using Students t-test. Average values were obtained from recordings twenty seconds in duration obtained prior to the first intervention (either umbilical cord clamping or initiation of ventilation) and throughout the intervention and selected time points. All subsequent data were compared over time and between groups using a two-way repeated measures ANOVA for postnatal physiological data with post-hoc analysis (Holm-Sidak) determining the time that differences were evident (Sigmastat v3.0, SPSS Inc.). Data are presented as mean ± SEM unless otherwise stated. Statistical significance was accepted for p<0.05.

## Results

### Fetal characteristics

Fetal body weights were not different between groups (Mean (SD): Vent 1^st^: 3.6 ± 0.6 kg: Clamp 1^st^: 3.2 ± 0.4 kg;). There were more males in the Clamp 1^st^ group (Table 1), but we have previously shown no sex-related differences to the cardiopulmonary haemodynamic transition at birth in preterm lambs[21]. Prior to any intervention, fetal pH tended higher, PaCO_2_ was significantly lower and PaO_2_ was significantly higher in Clamp 1^st^ lambs compared to Vent 1^st^ (Table 1); other fetal arterial blood gas parameters were not different between groups (Table 1). Fetal cerebral oxygen content (Table 1) was not different between groups prior to the intervention.

**Table 1:** Fetal Characteristics.

**Group**	**n**	**Male (%)**	**pH**	**PaCO_2_ (mmHg)**	**PaO_2_ (mmHg)**	**SaO_2_ (%)**	**Hb (g/dl)**	**CaO_2_**
Vent First	10	20	7.20 ± 0.03	65.8 ± 3.0	20.4 ± 2.6	63.2 ± 4.6	12.4 ± 0.5	10.1 ± 0.9
Clamp First	7	71	7.31 ± 0.02^#^	49.2 ± 4.8*	29.4 ± 2.4*	68.2 ± 4.7	11.3 ± 0.4	10.9 ± 1.0

Fetal blood gas values of pH, Partial pressure of arterial (Pa) oxygen (O_2_), carbon dioxide (PaCO_2_), haemoglobin (Hb) and arterial oxygen content (CaO_2_).

*indicates significant difference (p<0.05)

^#^ indicates trend (p<0.06).

### Ventilation and respiratory values

In Clamp 1^st^ lambs, ventilation commenced at a mean of 79 s (range 48–134 s) after umbilical cord clamping, which is greater than that recommended in international guidelines (< 60 s)[22]. In Vent 1^st^ lambs, ventilation commenced at a mean of 198 s (range 149–240 s) prior to umbilical cord clamping, which was the time that PBF was observed to increase, which is indicative of the haemodynamic transition.

**Fig 1:**
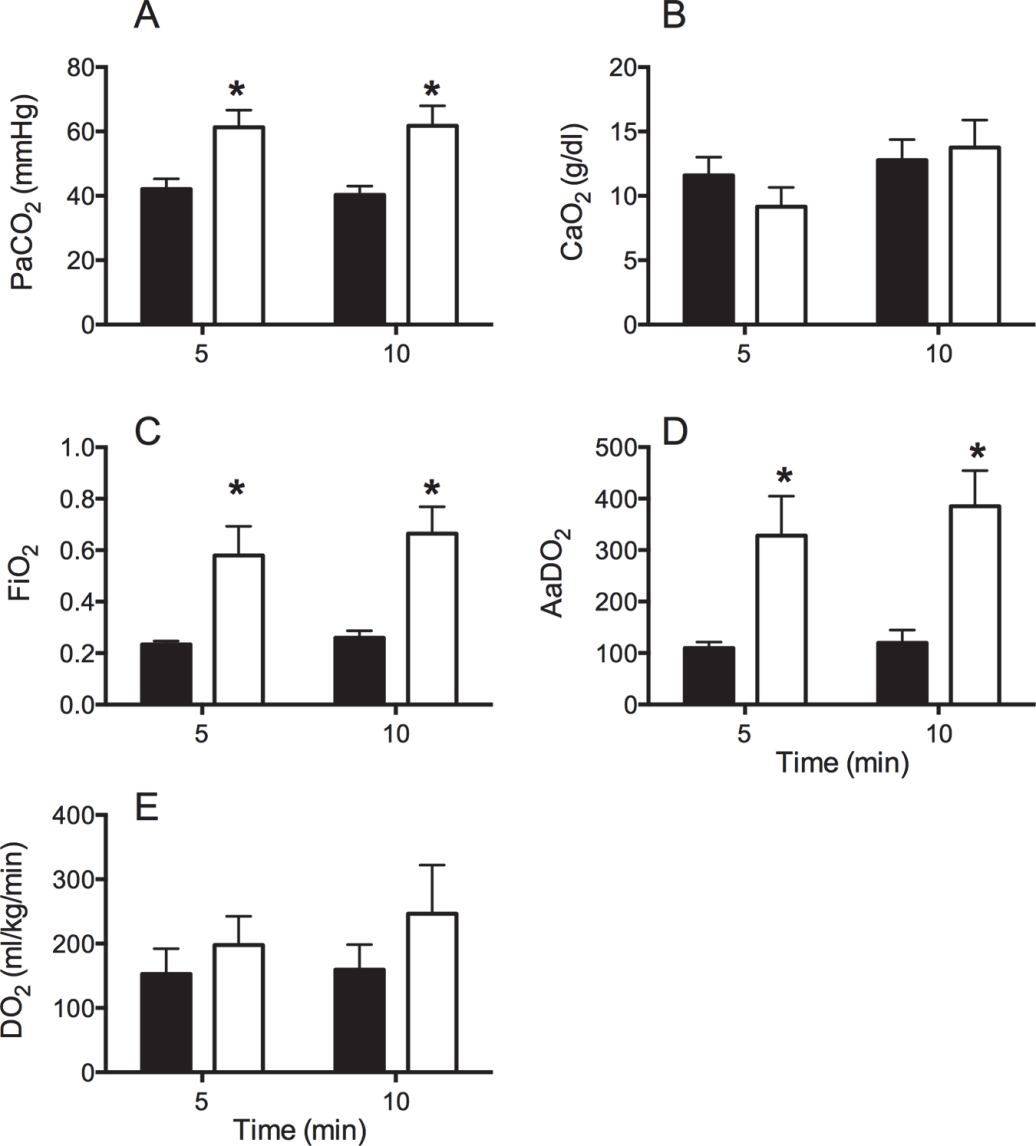
Blood gas parameters during ventilation. The partial pressure of (A) arterial carbon dioxide (PaCO_2_), (B) arterial oxygen content (CaO_2_), (C) the fraction of inspired oxygen (FiO_2_), (D) alveolar-arterial difference in oxygen (AaDO_2_) and (E) cerebral oxygen delivery (DO_2_) in Vent 1^st^ (Black) and clamp 1^st^ (white) preterm lambs. * indicates significant difference Vent 1^st^ vs. Clamp 1^st^ (p<0.05). Vent 1^st^ and Clamp 1^st^ lambs had similar arterial oxygen content and cerebral oxygen delivery, but Clamp 1^st^ lambs had worse PaCO_2_ and AaDO_2_ and thus required higher FiO_2_ to achieve similar tissue levels 5 and 10 minutes after delivery.

Tidal volume, peak inspiratory pressure, mean airway pressure and respiratory rate were not different between groups throughout the 10 min of ventilation (Data not shown). pH tended to be higher in Vent 1^st^ lambs 5 and 10 minutes after initiation of ventilation (p = 0.053). PaCO_2_, oxygenation index and AaDO_2_ were significantly lower in Vent 1^st^ lambs compared to Clamp 1^st^ lambs (Fig. 1), indicative of better oxygenation. PaO_2_ was not different between groups at 5 or 10 min. Vent 1st lambs received significantly lower FiO_2_ at 5 min (Mean (range) Vent 1st: 0.23 (0.21–0.32) vs. Clamp 1st: 0.58 (0.21–1.0); p<0.005) and at 10 min (Vent 1st: 0.26 (0.21–0.48) vs. Clamp 1st: 0.66 (0.21–1.0); p<0.001) (Fig. 1).

**Fig 2:**
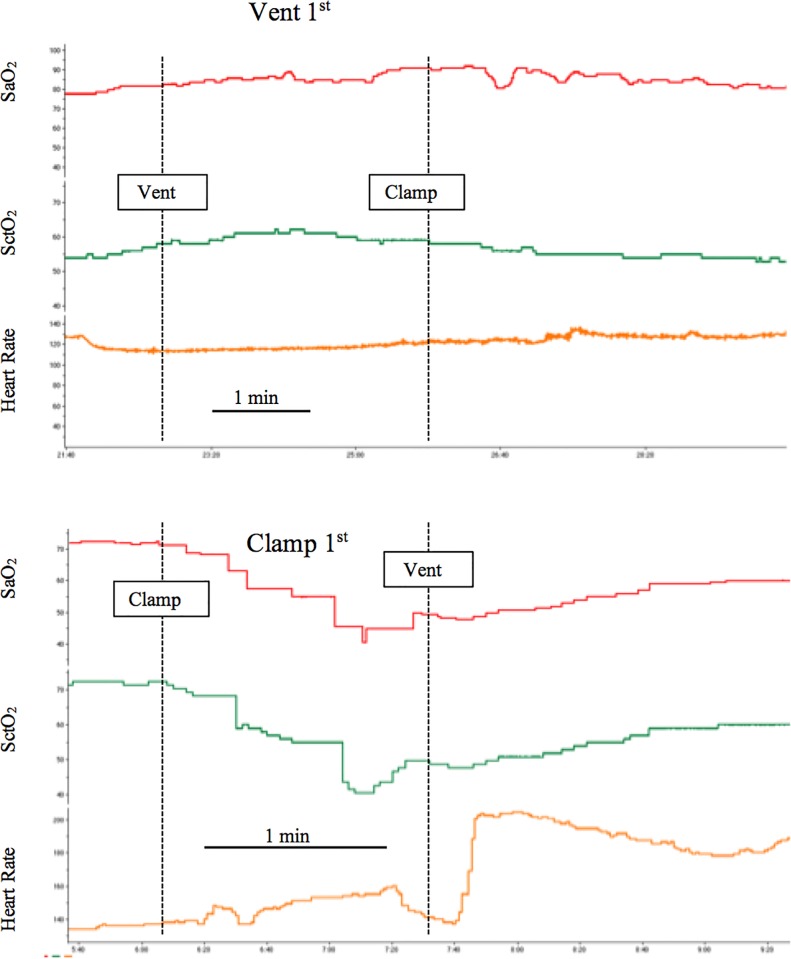
Effect of the timing of ventilation onset relative to umbilical cord clamping. Representative traces obtained from a lamb in which ventilation was initiated prior to umbilical cord clamping (Vent 1^st^), and a lamb in which umbilical cord clamping was conducted prior to the initiation of ventilation (clamp 1^st^). Dashed line indicates when an intervention occurred as labeled on the graphs. Note the difference in time scale. SpO_2_—arterial oxygen saturation, SctO_2_—cerebral oxygenation.

**Fig 3:**
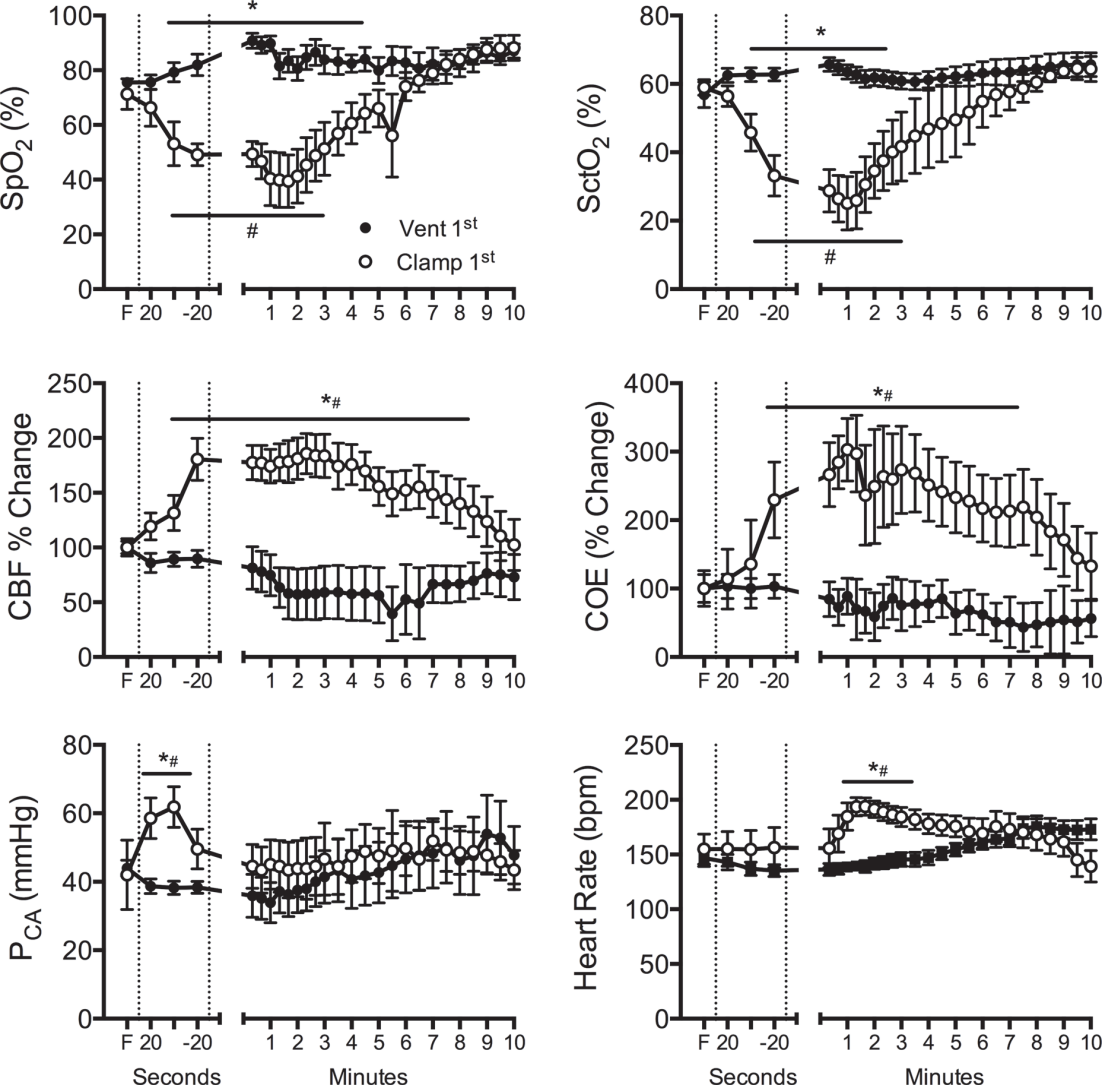
Arterial and cerebral oxygen saturation and haemodynamics. (A) Arterial saturation of oxygen measured by pulse oximetry (SpO_2_), (B) cerebral oxygenation (SctO_2_), (C) cerebral blood flow (CBF), (D) cerebral oxygen extraction (COE), (E) arterial pressure measured in a carotid artery (P_CA_) and (F) heart rate measured in Vent 1^st^ (closed circles) and Clamp 1^st^ (open circles) preterm lambs. First dashed line indicates ventilation onset for Vent 1^st^ lambs or umbilical cord clamping for Clamp 1^st^ lambs; second dashed line indicates umbilical cord clamping for Vent 1^st^ lambs and ventilation onset for Clamp 1^st^ lambs. F = fetal value. * indicates significant difference Vent 1^st^ vs. Clamp 1^st^ (p<0.05). # indicates time difference from fetal (F) value (p<0.05). In the time between umbilical cord clamping and ventilation onset, Clamp 1^st^ lambs significantly reduced arterial and cerebral oxygen saturation, and increased CBF and oxygen extraction to compensate. Ventilation onset increased arterial and cerebral oxygen saturation back to Vent 1^st^ lambs values by 5 min. Vent 1^st^ lambs maintained steady oxygenation and haemodynamics during the same time.

A representative real-time recording illustrating the effect of the timing of ventilation onset relative to umbilical cord clamping on arterial and cerebral oxygenation and cerebral blood flow is shown in Fig. 2. In Vent 1^st^ lambs, there was a steady increase in SpO_2_ with no discernible effect on SctO_2_, CBF or blood pressure (Fig. 3). Subsequent clamping of the umbilical cord had little physiological effect.

Clamping of the umbilical cord prior to ventilation resulted in a decrease in arterial saturation and cerebral oxygenation (Fig. 3). Prior to the initiation of ventilation and after clamping of the cord, Clamp 1^st^ lambs also showed a rapid increase in systolic pressure, cerebral blood flow and cerebral oxygen extraction (Fig. 3). In the time taken between cord clamping and ventilation onset in the clamp 1^st^ group, SpO_2_ fell from 68% to 29% (range of fall in SaO_2_ 11–62%) and SctO_2_ fell from 52–31% (range of fall in SctO_2_ 19–57%). Initiation of ventilation in Clamp 1^st^ lambs resulted in a rapid increase in heart rate, which remained significantly higher than Vent 1^st^ lambs until 5 minutes (Fig. 3). SaO_2_ and SctO_2_ increased, and reached values similar to Vent 1^st^ lambs by 5–6 minutes (Fig. 3). CBF and cerebral oxygen extraction fell to similar levels as Vent 1^st^ lambs by 9 minutes. Arterial oxygen content and oxygen delivery in the carotid artery were not different between groups at 5 and 10 min (Fig. 1). Blood pressure rapidly returned to baseline values by 1 min and was not different between groups thereafter.

## Discussion

Aeration of the lung at birth is critical for the successful transition from a fetus to a newborn. Pulse oximetry is used extensively to assess the progress of the cardio-respiratory transition in preterm infants and guides interventions including supplemental oxygen delivery and respiratory support. We investigated whether the establishment of ventilation prior to umbilical cord clamping, improves arterial and cerebral oxygenation during the transition at preterm birth. Our findings demonstrate that ventilation before umbilical cord clamping improves arterial and cerebral oxygenation and haemodynamics, while cord clamping before adequate ventilation results in rapid arterial and cerebral desaturation. These findings suggest that the timing of ventilation onset relative to umbilical cord clamping may play a critical role in subsequent oxygenation of the preterm infant in the delivery room.

International, national and unit guidelines for the use of supplemental oxygen are informed by published SpO_2_ nomograms[6]. These allow clinicians to target specific SpO_2_ levels during the first minutes after birth. The use of SpO_2_ nomograms has led to numerous clinical trials investigating the best way to reach these targets in the delivery room, including the use of automated devices (e.g. [23]). An interesting finding of these nomograms is that the median SpO_2_ at 1 minute is similar to the normal fetal values, meaning that half of these otherwise healthy infants may be considered to be hypoxic at this time point. Our findings demonstrated that the period of time between cord clamping and onset of ventilation, during which time the apnoeic preterm lamb was being dried, warmed and positioned on the infant warmer, resulted in a more than halving of arterial and cerebral oxygen saturation (Figs. 2 & 3). This occurred despite having a higher PaO_2_ and lower PaCO_2_ than Vent 1^st^ lambs immediately prior to umbilical cord clamping. These findings demonstrate the potential importance of adequate ventilation or breathing prior to umbilical cord clamping for maintenance of oxygenation.

Subsequent ventilation of Clamp 1^st^ lambs resulted in a slow improvement in oxygenation, despite similar tidal volumes and pressures to the Vent 1^st^ animals. It took 5–6 minutes before SpO_2_ was within “normal” ranges based on the Dawson nomogram. These findings are of concern given the known association between prolonged episodes of hypoxia and preterm brain injury[24]. As a consequence, Clamp 1^st^ lambs required a significantly higher FiO_2_ to reach the target SpO_2_. The higher requirement for FiO_2_ in the Clamp 1^st^ lambs directly parallel the transient requirement for FiO_2_ of preterm infants in clinical trials studying the effect of the initial FiO_2_ on systemic oxygenation[8,25,26]. In these trials, the mean FiO_2_ at 5 min for most preterm infants, irrespective of the starting FiO_2_, was between 50–60%, consistent with our observations. All infants in these trials underwent immediate cord clamping. The transient nature of the increased oxygen requirement in the delivery room has been a consistent observation in preterm infants after immediate cord clamping. Our study suggests that the cause of the increased requirement for FiO_2_ in preterm infants may be due to a poor cardiovascular transition resultant from inadequate aeration of the lung prior to umbilical cord clamping. Further, the level of FiO_2_ delivered in our study has been shown to produce local pulmonary and systemic changes indicative of oxidative stress in preterm infants[25,27], which can lead initiate lung injury resulting in longer/higher oxygen requirements in the NICU. Oxidative stress is also a major cause of preterm perinatal brain injury[24,28]. Although we did not measure markers of brain inflammation and oxidative stress in this study, ventilation prior to umbilical cord clamping may reduce these pathways.

The reference ranges for regional cerebral tissue oxygen saturation have recently been established in normal term and preterm infants, and, consistent with the Dawson nomogram, cerebral oxygen saturation was low at 2 minutes at normalised by 5–6 minutes[10]. In our study we observed a similar fall in SctO_2_ as SaO_2_ in our clamp first group, which took 5–6 minutes to normalise after ventilation onset. In our hands regional oxygen saturation is lower in preterm lambs compared to humans[29]. This difference likely relates to species differences, location of the probes or the different devices used (INVOS vs. CASMED). Irrespective, ventilation onset prior to cord clamping prevented this fall in cerebral oxygen saturation. Given that animal studies have shown that an average cerebral tissue oxygenation index of less than 55% results in cerebral injury [30], delaying cord clamping until ventilation onset may prevent prolong periods of time below this critical threshold.

CBF increased rapidly, by ∼80% within the first 60 seconds after cord clamping in Clamp 1^st^ lambs and remained elevated throughout the entire ventilation strategy compared to Vent 1^st^ lambs (see Fig. 3). The rapid increase in CBF we observed likely occurs through a combination of haemodynamic responses to umbilical cord clamping (removal of the capacitance placental circulation), and a subsequent increase due to an increasingly hypoxic environment. The later suggests intact cerebral autoregulation in lambs of this gestation, which allowed them to maintain cerebral oxygen delivery (See Fig. 1B). However, preterm infants are known to have immature cerebral vascular beds, which are prone to leakage and haemorrhage[31] and have impaired ability to autoregulate CBF, particularly during the first days of life[32]. Abnormal fluctuations in CBF (defined as prolonged swings in CBF, either high or low, for greater than 10 to 20s)[33] is a major mechanism of preterm brain injury[24,28,34]. The rapid rise in CBF we observed in this study, may translate to an increased the risk of cerebral vascular leakage and/or haemorrhage in preterm infants.

Past and current trials of delayed cord clamping use time after delivery (from 30 s up to 3 min) to determine when the umbilical cord is clamped[35]. The use of time is based on the idea that the main benefit of delaying cord clamping is to maximize blood volume within the infant (increase placental to fetal transfer). Our recent research suggests that an additive benefit of delaying cord clamping may be the establishment of breathing prior to clamping[15]. This supports the clinical findings of improved cardiovascular stability in preterm infants after delayed cord clamping[16,17,36], albeit at a time-point much later than we are studying (6–48 h), and the finding that respirations are an important determinant of the volume of placental transfusion[37].

Aeration of the lung, whether it occurs via spontaneous breathing or mechanical ventilation, is the critical factor causing the decrease in pulmonary vascular resistance and increase in pulmonary blood flow at birth, which initiates the cardiopulmonary transition[2,4,38]. During fetal life the majority of left ventricular preload and fetal oxygenation is provided by placental blood flow. Decreasing pulmonary vascular resistance at birth, increases pulmonary blood flow and allows for the pulmonary circulation to supply left ventricular preload and to replace the placental oxygenation supply with a pulmonary oxygen supply. This maintains cerebral circulatory stability throughout delivery[15]. For this reason we used the increase in pulmonary blood flow to indicate successful cardiopulmonary transition and to dictate the timing of cord clamping in Vent 1^st^ lambs. Measurement of pulmonary blood flow during the transition is difficult to do in clinical practice, thus using aeration of the lung, as indicated by vigorous breathing of the baby, is the appropriate pseudo marker of transition. However, the influence of ventilation onset relative to the timing of umbilical cord clamping on systemic and cerebral oxygenation was not known. Our findings, and those of our previous study, suggest that the timing of umbilical cord clamping should not be based on elapsed time after birth, but rather focus on the physiology of the newborn, in particular on adequate breathing efforts. Indeed, a recent review stated that “rapid assessment of the newly born infant and the initial steps of drying, providing warmth, clearing the airway, and providing specific stimulation to breathe can be carried out with the umbilical circulation intact”, which would require a paradigm shift in our current clinical practice[36]. Our study also raises the potential issue that the current oxygen saturation nomograms with a dip in oxygenation below fetal levels in the first minutes of life are an artefact of immediate cord clamping, a potentially abnormal event, and are not evident when ventilation is established prior to cord clamping. Indeed, a recent study showed a higher SaO_2_ and lower heart rate in the first 3 minutes in infants who underwent delayed cord clamping and skin to skin contact[39]. Although no evaluation of the efficacy of breathing was undertaken, these were uncompromised term infants and started breathing within seconds after birth.

There are limitations to our study that may alter the clinical context. In our study, lambs were delivered via caesarean delivery while the ewe was receiving ventilation under anaesthesia. While this allowed us to demonstrate the importance of the timing of ventilation onset relative to umbilical cord clamping in a controlled situation, it does have limitations in transition to the delivery room. All lambs were completely exteriorised from the uterus for ∼ 181 s prior to either cord clamping or ventilation onset. This was conducted to allow for stabilisation of cardiopulmonary haemodynamics prior to intervention. While this could be interpreted as delayed cord clamping, the influence of maternal and fetal anaesthesia preventing fetal respiratory efforts, coupled with the absence of labour, prevents comparison with clinical delayed cord clamping. Indeed, we did not observe any change to haemodynamics or oxygenation during the EXIT procedure. In the clinical scenario, many factors including labour and/or oxytocin administration are likely to have significant effects on umbilical blood flow, cardiopulmonary haemodynamics and oxygenation[40] but these were not assessed in this study.

Vaginal delivery is known to result in a higher SaO_2_ in the first minutes of life [6]. Our studies have not investigated the influence of ventilation onset prior to cord clamping in vaginally delivered preterm lambs, which may alter the oxygenation response. However, we would hypothesise that delaying umbilical cord clamping until breathing is established has the most benefit in caesarean delivered neonates, due to the increased volume of lung liquid required to be removed[41,42], and the reduced likelihood of spontaneous breathing compared to vaginally delivered infants. More studies are required to investigate this.

It is important to note that the lambs in this study were not breathing. A significant proportion of preterm infants make some breathing efforts, gasping or crying, during delivery[43]. Thus the fall in systemic and cerebral oxygenation we observed might more closely mimic that seen clinically in an apnoeic infant, or an infant making poor respiratory efforts, compared to an infant making appropriate spontaneous breathing efforts. However, it is pertinent to note that ∼19% of all babies born in Australia require some respiratory support (oxygen or IPPV) to reach the target SpO_2_ nomogram[5], which indicates that a large proportion of otherwise normal babies are not transitioning smoothly at birth. The severity of the fall in systemic and cerebral oxygenation after birth is thus dependent upon the degree of aeration of the lung, which is best determined clinically by the degree of breathing.

The mean time between umbilical cord clamping and ventilation onset in the clamp 1^st^ group of 79 s was longer than the recommended time for initiation of respiratory support (< 60 s)[22] and that reported in an audit of the time for initiation of respiratory support in preterm infants (70 (23) s)[44]. While this appears to be a long time, the time it takes to transfer the infant from the delivery table to the resuscitation area, perform initial assessment, begin warming, clear airways and initiate adequate respiratory support all takes significant time, and in some instances the initiation of respiratory support in preterm infants is occurring beyond this time-point[44].

## Conclusions

In summary, the initiation of ventilation prior to umbilical cord clamping in preterm lambs resulted in smoother systemic and cerebral oxygenation during transition. Our study suggests that the onset of breathing/aeration of the lung is an important determinate of SpO_2_ and SctO_2_ within the first minutes of life. Physiological-based umbilical cord clamping (delaying until ventilation is established) may improve short and long term outcomes in preterm infants.
